# Use patterns, use values and management of *Afzelia africana* Sm. in Burkina Faso: implications for species domestication and sustainable conservation

**DOI:** 10.1186/s13002-018-0221-z

**Published:** 2018-03-27

**Authors:** Larba Hubert Balima, Blandine Marie Ivette Nacoulma, Marius Rodrigue Mensah Ekué, François N’Guessan Kouamé, Adjima Thiombiano

**Affiliations:** 1 West African Science Service Center on Climate Change and Adapted Land Use (WASCAL) Graduate Research Program on Climate Change and Biodiversity, Unité de Formation et de Recherche Biosciences, Université Félix Houphoüet Boigny, BP 165, Abidjan 31, Côte d’Ivoire; 2 Department of Plant Biology and Physiology, Laboratory of Plant Biology and Ecology, University Ouaga I Pr. Joseph Ki-Zerbo, 03 Box 7021, Ouagadougou 03, Burkina Faso; 3 Bioversity International, Forest Genetic Resources and Restoration Team, Box 2008 (Messa), Yaoundé, Cameroon

**Keywords:** African mahogany, Local knowledge, Sustainability, Sahel, West Africa

## Abstract

**Background:**

The lack of literature on the interactions between indigenous people and the valuable agroforestry trees hinder the promotion of sustainable management of plant resources in West African Sahel. This study aimed at assessing local uses and management of *Afzelia africana* Sm. in Burkina Faso, as a prerequisite to address issues of domestication and sustainable conservation.

**Methods:**

One thousand forty-four peoples of seven dominant ethnic groups were questioned in 11 villages through 221 semi-structured focus group interviews. The surveys encompassed several rural communities living around six protected areas along the species distribution range. Questions refer mainly to vernacular names of *A*. *africana*, locals’ motivations to conserve the species, the uses, management practices and local ecological knowledge on the species. Citation frequency was calculated for each response item of each questionnaire section to obtain quantitative data. The quantitative data were then submitted to comparison tests and multivariate statistics in R program.

**Results:**

*A*. *africana* is a locally well-known tree described as a refuge of invisible spirits. Due to this mystery and its multipurpose uses, *A*. *africana* is conserved within the agroforestry systems. The species is widely and mostly used as fodder (87.55%), drugs (75.93%), fetish or sanctuary (70.95%), food (41.49%), and raw material for carpentry (36.19%) and construction (7.05%). While the uses as fodder, food and construction involved one organ, the leaves and wood respectively, the medicinal use was the most diversified. All tree organs were traditionally used in 10 medical prescriptions to cure about 20 diseases. The species use values differed between ethnic groups with lower values within the Dagara and Fulani. The findings reveal a total absence of specific management practices such as assisted natural regeneration, seeding, or transplantation of *A*. *africana* sapling. However, trees were permanently pruned and debarked by local people. Harvesting of barks mostly contributed to the decline of the species populations. Local people acknowledged declining populations of *A*. *africana* with lower densities within the agroecosystems. They also perceived between individuals, variations in the traits of barks, leaves, fruits and seeds. Significant differences were found between ethnic groups and gender regarding the species uses. Local knowledge on the species distribution differed between ethnic groups.

**Conclusion:**

This study showed the multipurpose uses of *A*. *africana* throughout Burkina Faso*.* The results provide relevant social and ecological indicators to all stakeholders and constitute a springboard towards the species domestication and the elaboration of efficient sustainable conservation plans.

## Background

In the Sahelian countries, most people living in rural areas rely on wild plant products for their daily subsistence [1–4]. The traditional plants use worldwide represents an invaluable reservoir of knowledge and a large potential of yet undiscovered use of natural resources [5]. In fact, a frequent use of wild plants and a transmission of knowledge from generation to generation give indigenous people a profound knowledge of plant resources in the local environment [6]. Local knowledge relates to the useful species, traditional plant uses and forestry management practices [5, 6]. Such informations are incontestably fundamental for any management strategies aimed at sustainable conservation of plant resources [3, 6]. Therefore, local knowledge can complement scientific data in the global frame of biodiversity management.

Ethnobotanical studies conducted in West Africa reported the uses of forest products by indigenous people to satisfy their daily needs [1, 3–5]. However, a little attention has been paid to the interactions between farmers and agroforestry trees within the agroecosystems. Yet, agroforestry systems provide ecosystem services and reduce human impacts on natural forests [7], and therefore contribute to carbon sequestration [8] and biodiversity conservation [9]. Indeed, agrobiodiversity contributes to improve local people incomes, food security and nutrition [10]. However, the current coupled effects of climate change and land cover change are leading to an increasing habitat loss for many useful plants [5] reducing their goods and services. Therefore, it is important to valorize local valuable plants in order to achieve their domestication and their sustainable conservation in the agroforestry systems. The valorization and domestication of plants require scientific approach based on indigenous knowledge on their use forms and use values and locals’ conservation strategies and perceptions of species variation.

*Afzelia africana* Sm. (called African mahogany or African oak) is an African legume tree species with high socio-economic, industrial, cultural and ecological importance [11]. The species belongs to the Fabaceae-Caesalpinioideae family and is distributed in the West African savannahs and forests ranging from the Sudanian Regional Centre of Endemism to the Guineo-Congolean Regional Centre of Endemism [11, 12]. *A*. *africana* leaves are used in human feeding and constitute forage for livestock [13–15]. Moreover, the wood is overexploited and commercialized for various industrial needs [11] while the barks abound in various medicinal properties [12, 16]. *A*. *africana* embodies also spiritual values for some ethnic groups in West Africa [11, 17]. Studies revealed that the natural regeneration of this species is low in Burkina Faso [18, 19]. *A*. *africana* is also facing an overexploitation by communities across West African countries [14, 20, 21] resulting in a decline of its natural populations. Therefore, it is considered as a threatened species in many countries [20, 22, 23].

Over the last decade, several studies evaluated local knowledge on the valuable woody plant species of West Africa [3, 24–27]. These studies showed how location [27], ethnicity [3, 26], gender [24] and age [3, 25] influence the uses of plant resources. Despite the threats reported by some authors on the species [14, 20–22], few ethnobotanical data are available on *A*. *africana*. The use forms, use values, management practices and local perceptions on the dynamics and morphological variations of this species are undocumented. Such data are however prerequisites to address the species valorization and domestication issues.

The study addressed these issues within several rural communities living around six protected areas along the distribution range of *A*. *africana* in Burkina Faso. These surrounding peoples encompass both autochthons and migrants of seven dominant ethnic groups of different cultural backgrounds. We aimed at assessing the use forms, use values, management practices and local ecological knowledge on the species for its more reasonable use and sustainable conservation. Indeed, understanding how rural communities use and manage plant resources, and analyzing their ecological knowledge on the species, and the drivers behind, are indispensable supports towards the species sustainability.

The following research questions were addressed: (i) What are the use forms of *A*. *africana* and how are the use values distributed across ethnic groups? (ii) What are local management practices and their impacts on the species survival? (iii) How do local people perceive the spatiotemporal dynamics and the morphological variations of the species? (iv) Which factor does affect local uses and ecological knowledge on the species? Given the wide distribution range of *A*. *africana* in the country and the difference in peoples’ cultural backgrounds, we firstly assumed that local people have a greater knowledge on the species uses and that the species utilitarian value differs between ethnic groups. Secondly, we assumed that as both multipurpose [13, 15, 16] and threatened species [14, 22] along its distribution range, locals have developed various management strategies to ensure the species long-term use, but the harvesting patterns of organs affect negatively the species survival. We thirdly assumed that local people perceive the regressive dynamic of *A*. *africana* and morphological variations of its individuals in their environment. We finally assumed that as magic tree [11, 17], local uses and ecological knowledge on *A*. *africana* are gender specific.

## Methods

### Study area

**Fig. 1:**
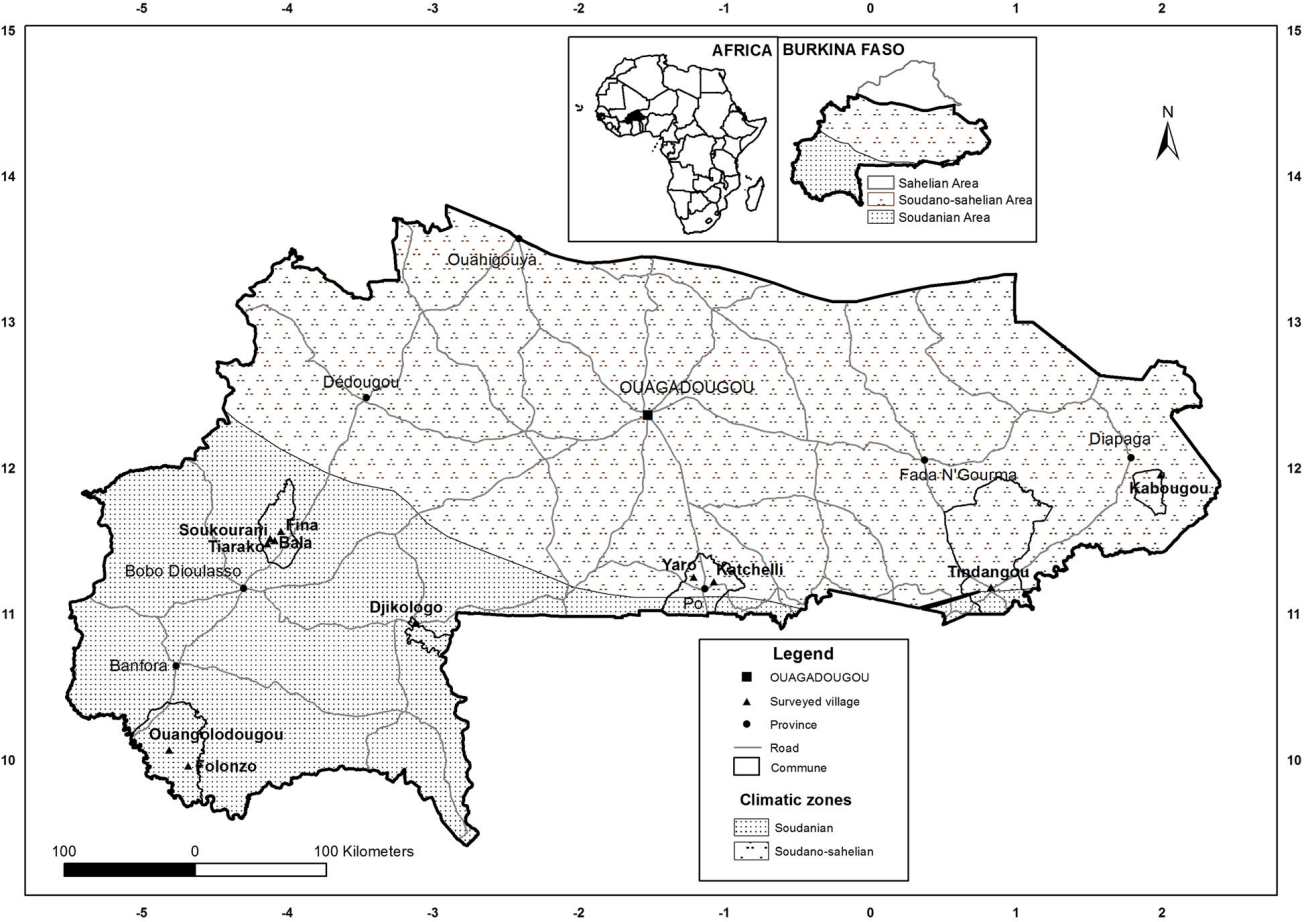
Localization of the study area in Burkina Faso. No legend (embedded on the map)

The study was carried out at the Sudano-sahelian and the Sudanian climatic zones of Burkina Faso which represent the distribution area of *A*. *africana* in the country. The sites were Kabougou, Tindangou, Katchelli and Yaro in the Sudano-sahelian zone and Djikologo, Folonzo, Ouangolodougou, Fina, Bala, Tiarako and Soukourani in the Sudanian zone (Fig. 1). These villages were selected around six protected areas (PAs) which shelter natural populations of *A*. *africana*. In the Sudano-sahelian zone, the protected areas consisted of the W National Park and the Singou Reserve located in Eastern Burkina Faso, and the National Park Kaboré Tambi in Center-Southern. The Wildlife Reserve of Bontioli (South-Western), the Total Reserve of Comoé Leraba and the Biosphere Reserve of Hippopotamus Pool (Western) were selected in the Sudanian zone. In the Sudano-sahelian zone, the annual rainfall varies between 600 and 900 mm over 4 to 5 months while the annual temperature ranges from 20 to 30 °C. The vegetation is composed of a mosaic of various savannah types (shrubs, tree and wooded savannahs) and forests. The Sudanian zone has annual rainfall between 900 and 1100 mm with a rainy season of 5 to 6 months. This area is characterized by low temperature (20–25 °C). The vegetation consists mainly of savannahs, dry forests and gallery forests [28]. The species are typically Sudano-sahelian in the Sudano-sahelian zone and Sudano-Guinean in the Sudanian one. Rural populations of the study sites are mainly farmers and herders.

### Sampling design and ethnobotanical surveys

Whether rural communities are known to have profound knowledge on plant resources [6], people bordering the PAs are the most knowledgeable regarding the threatened plants from which the PAs remain the main refuges. Based on the nationwide distribution range of *A*. *africana*, six natural populations (three per climatic zone) of the species were selected in the PAs. Around these PAs, the nearest 11 villages (surrounding villages) were sampled. Seven ethnic groups were finally selected in the villages by considering their dominance and people residence status. People origin or residence status hereby refers to autochthon and migrant. The five dominant ethnic groups of the study sites and the two most important ethnic groups of Burkina Faso were sampled. In the villages of the Sudano-sahelian zone, the autochthon groups Gourmantché and Kasséna were selected. The autochthons Dagara, Bobo and Dioula were sampled in the Sudanian zone given the high cultural diversity of this part of the country. In both climatic zones, the selected migrant groups were Mossi and Fulani. The five autochthon groups represented  the most dominant ethnic groups of the study sites. The Mossi and Fulani (migrants) constitute the two most dominant ethnic groups of Burkina Faso [3].

A three-way factorial design of two levels of residence status, seven levels of ethnicity and two levels of gender was employed to select focus groups through a random stratified scheme. Semi-structured focus group interviews were adopted because this survey method confronts directly many informants and enables to record only reliable and unbiased data. Overall, 679 men and 365 women were separately interviewed in 135 and 86 focus groups respectively, each group spanning 5–10 peoples. This unbalanced sampling size between men and women was due to the fact men were more available and more willing to be interviewed on the species than women. Given that young peoples usually lack experience [3], surveys were limited to adult persons (at least 30 to 40), so that only experienced informants were interviewed. Informants were questioned about local names of the species and their meanings, the motivations to conserve *A*. *africana* in the farmlands, the species uses, traditional management practices, and local ecological knowledge on the species.

### Data analysis

At each question section, informants’ responses were grouped into answers’ groups according to their similarities. These answers were then converted into presence absence matrix of 1 or 0 (signifying whether an informant cites an item or not) [3] comprising informants’ ethnic group and gender as line variables, and the responses derived from the aforementioned questions as column variables. The ethnobotanical indicators analyzed through the dataset were the citation frequencies, the fidelity level, the species use values and the index value of organs (Table 1). For each question section, citation frequency was calculated for each response level to convert the presence absence matrix into quantitative data. The frequency of response or citation is defined as the proportion between the number of positive answers (number of citations) for each questionnaire item and the total number of informants.

**Table 1:** Ethnobotanical indicators analyzed through the dataset

Questions	Items’ groups	Variables	Factors	Indices
Motivations to conserve	Agroforestry tree	FL, *F*	EG, G	
	Sacred or magic			
	Multiple uses			
	Threats			
Use categories	Food	FL, *F*	EG, G	Use value per EG
	Medicinal			
	Fodder			
	Culture			
	Firewood			
	Construction			
Organs exploited	Leaves	*F*	EG, G	Index value of organ
	Barks			
	Wood			
	Fruits			
	Seeds			
Local practices	Natural RGN	FL, *F*	EG, G	
	Assisted RGN			
	Seeding			
	Transplantation			
	Pruning			
	Debarking			
	Fire protection			
Occurrence habitats	Protected areas	FL, *F*	EG, G	
	Sacred groves			
	Fallows			
	Farmlands			
	Human settlements			
Species status	Abundant	FL, *F*	EG, G	
	Rare			
Variations criteria	Tree size	FL, *F*	EG, G	
	Tree density			
	Tree morphology			
Existence of ecotypes	Yes/no	*F*	EG, G	

*F* citation frequency, *FL* fidelity level, *EG* ethnic group, *G* gender, *RGN* regeneration

To determine the species uses, the recorded usages were arranged by use category and the frequency of each use category was computed. The fidelity level representing the degree of consensus between informants was calculated for each use category and used across the ethnic groups to measure the distribution of the species uses among informants.

The fidelity level (FL) was computed as followed [10]:

$$ \mathrm{FL}=\mathrm{Ip}/\mathrm{Iu}\times 100\% $$

where Ip represents the number of respondents answering positively to each question item and Iu the total number of positive answers for each question section.

The index value of organs (IVO) was used to determine the most exploited organs. This index was computed using the formula:

$$ \mathrm{IVO}=\sum {N}_{\mathrm{u}i}\times \raisebox{1ex}{$1$}\!\left/ \!\raisebox{-1ex}{${N}_{\mathrm{tu}}$}\right. $$

where *N*_u*i*_ is the number of uses patterns of each organ quoted by informants *i* and *N*_tu_ the total number of uses of all organs quoted by the informants *N*.

The IVO were compared between organs using the parametric paired *T* test.

The usefulness of *A*. *africana* expressed by its use values (UVs) was assessed for each ethnic group using the following formula [29]:

$$ \mathrm{UVs}=\sum {\mathrm{UV}}_{is}\times \raisebox{1ex}{$1$}\!\left/ \!\raisebox{-1ex}{${N}_s$}\right. $$

where UV_*is*_ equals to the use value of the species *s* for an informant *i* and *N*_*s*_ the total number of persons interviewed for the species *s*.

The UVs were also compared between ethnic groups through the paired *T* test to determine which ethnic groups used mostly the species.

The management practices developed by locals were analyzed via the fidelity level.

Local ecological knowledge on the species were also assessed using the fidelity for such questionnaire item.

To identify the drivers of people knowledge on *A*. *africana*, multivariate statistics and comparison tests were performed. The matrix of frequencies of all responses was first submitted to principal component analysis (PCA) to determine the links between people knowledge and their ethnic-gender memberships. Thus, the seven ethnic groups were divided into 14 subgroups by considering the gender. The 14 subgroups were then codified using the three first letters of the corresponding ethnic group and the first letter of the gender [10]. Finally, the non-parametric Kruskal-Wallis test was performed to seek for significant differences in local knowledge with regard to ethnicity and gender. All the statistical analyses were performed using R program (*R version 3.2.2*).

## Results

### Local names of *A*. *africana*

*A*. *africana* is identified through different names according to the ethnic groups (Table 2). Overall, these names referred to the inhabitants of the species known as "evil doing spirits", and the associated mystery or enigma. The names also related to the high fodder value of *A*. *africana* and the high resistance of its wood.

**Table 2:** Local names and their meanings

Ethnic group	Local names	Meanings
Bobo	Kibi	Dwarfs’ refuges, sacred species
Dagara	Kakala, kontontiè, kontontiénou	Dwarfs’ refuges, dangerous species
Dioula	Linguè	Invisible spirits, sacred species
Gourmantché	Bu nakpambu/kpambu, Linakpanli	Sacred, fodder species, sanctuary
Kasséna	Kolo	Invisible spirits, dangerous species
Mossi	Kakalga, kankalga	Invisible forces, dwarfs, imps’ refuge
Fulani	Kakalgahi, Panbouhi, Linguèhè	High fodder species, resistant wood

### Local motivations and use categories

African mahogany is conserved within the traditional agroforestry systems for various reasons. The main reasons include its sacredness to rural communities, its agroforestry potential and its multipurpose uses (Table 3).

**Table 3:** Local knowledge on *A*. *africana* in Burkina Faso

Criteria	Proposed variant	Bobo		Dagara		Dioula		Gourmantché		Kasséna		Mossi		Fulani	
		(*n* = 37)		(*n* = 40)		(*n* = 39)		(*n* = 29)		(*n* = 40)		(*n* = 16)		(*n* = 20)	
		*F*	FL	*F*	FL	*F*	FL	*F*	FL	*F*	FL	*F*	FL	*F*	FL
Motivations to conserve	Agroforestry	16	55.17	23	63.89	28	77.78	3	25	20	55.56	1	33.33	3	50
	Culture	6	20.69	7	19.44	6	16.67	7	58.33	14	38.89	1	33.33	2	33.33
	Multipurpose uses	5	17.24	6	16.67	2	5.56	1	8.33	2	5.56	1	33.33	1	16.67
	Threats	2	6.90	0	0	0	0	1	8.33	0	0	0	0	0	0
	Σ*F*	29	–	36	–	36	–	12	–	36	–	3	–	6	–
Use categories	Food	10	7.09	0	0	14	9.59	19	17.59	39	27.86	15	25.86	3	5.36
	Medicine	37	26.24	38	31.67	30	20.55	28	25.93	32	22.86	10	17.24	8	14.29
	Fodder	36	25.53	39	32.5	39	26.71	23	21.30	39	27.86	15	25.86	20	35.71
	Carpentry	16	11.35	5	4.17	27	18.49	17	15.74	1	0.71	11	18.97	10	17.86
	Culture	30	21.28	38	31.67	22	15.07	18	16.67	15	10.71	4	6.90	2	3.57
	Firewood	3	2.13	0	0	0	0	3	2.78	9	6.43	2	3.45	1	1.79
	Construction	4	2.84	0	0	8	5.48	0	0	1	0.71	0	0	4	7.14
	Shade	3	2.13	0	0	5	3.42	0	0	4	2.86	0	0	1	1.79
	Veterinary	1	0.71	0	0	0	0	0	0	0	0	1	1.72	7	12.5
	Fertilization	1	0.71	0	0	1	0.68	0	0	0	0	0	0	0	0
	Σ*F*	141	–	120	–	146	–	108	–	140	–	58	–	56	–
Species regeneration	Assisted	0	0	0	0	1	2.56	1	3.45	1	2.5	0	0	0	0
	Natural	37	100	40	100	38	97.44	28	96.55	39	97.5	15	100	20	100
	Sowing	0	0	0	0	0	0	0	0	0	0	0	0	0	0
	Transplantation	0	0	0	0	0	0	0	0	0	0	0	0	0	0
	Σ*F*	37	–	40	–	39	–	29	–	40		15	–	20	–
Local practices	Pruning	15	51.72	28	48.57	33	56.90	17	47.22	28	32.94	8	50	13	76.47
	Debarking	11	37.93	19	32.76	25	43.10	18	50	25	29.41	8	50	4	23.53
	Fire protection	3	10.34	11	18.97	0	0	1	2.78	32	37.65	0	0	0	0
	Σ*F*	29	–	58	–	58	–	36	–	85		16	–	17	–
Species status	Abundant	12	32.43	28	84.85	7	17.95	4	44.44	19	48.72	2	33.33	0	0
	Rare	25	67.57	5	15.15	32	82.05	5	55.56	20	51.28	4	66.67	9	100
	Σ*F*	37	–	33	–	39	–	9	–	39	–	6	–	9	–
Occurrence habitats	Farmlands	13	16.25	37	22.98	35	23.65	12	21.43	29	28.16	8	22.22	6	18.18
	Fallows	12	15	36	22.36	35	23.65	9	16.07	31	30.10	7	19.44	5	15.15
	Human settlements	7	8.75	18	11.18	15	10.14	4	7.14	0	0.00	3	8.33	1	3.03
	Protected areas	32	40	38	23.60	35	23.65	20	35.71	38	36.89	13	36.11	14	42.42
	Sacred groves	16	20	32	19.88	28	18.92	11	19.64	5	4.85	5	13.89	7	21.21
	Σ*F*	80	–	161	–	148	–	56	–	103	–	36	–	33	–
Variations criteria	Trees’ size	21	70	12	66.66	28	45.16	18	75	17	53.12	7	63.63	11	78.57
	Trees’ density	2	6.67	2	11.11	8	12.90	2	8.33	10	31.25	0		0	0
	Trees’ morphology	7	23.33	4	22.22	26	41.93	4	16.67	5	15.62	4	36.36	3	21.43
	Σ*F*	30	–	18	–	62	–	24	–	32	–	11	–	14	–

*n* number of interviewees per ethnic group, *F* frequency, *ΣF* sum of frequency per item, *FL* fidelity level

**Fig. 2:**
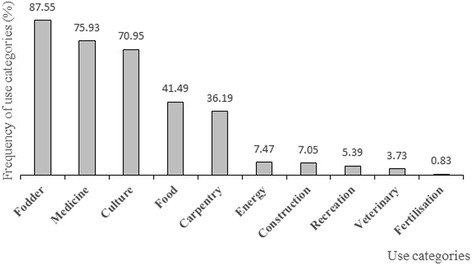
Use categories of *A*. *africana.* No legend

All the parts of *A*. *africana* were used for a wide range of purposes that can be grouped in ten main use categories (Fig. 2). The most important uses were fodder (87.55%), medicine (75.93%), culture (70.95%), and food (41.49%).

The fidelity levels (Table 3) highlighted the fodder use by all ethnic groups (21.30 < FL < 31.71), the medicinal use by the Bobo, the Dagara, the Dioula, the Gourmantché and the Kasséna (20.55 < FL < 31.67), the cultural use by the Bobo and the Dagara (21.28 < FL < 31.67), and the use as food by the Kasséna and Mossi (25.86 < FL < 27.86).

### Plant parts exploited and use values

*A*. *africana* parts exploited were roots, barks, seeds, fruits husk, leaves, aril and fruits. The index value of organs (IVO) showed that the leaves were the most exploited part, followed by wood and bark (Table 4). However, the seeds, the roots and the aril were less used.

**Table 4:** Comparison of index values of organs

Organs	IVO	*T* test
Barks	11.67	ab
Fruits	7.70	b
Fruit husk	0.48	b
Leaves	46.33	a
Seeds	4.69	b
Roots	6.50	b
Wood	22.50	ab
Aril	0.12	b

a*b: The levels non-connected by the same letters are significantly different

The use values revealed that *A*. *africana* spans mainly three to four use categories (2.80 ≤ UV ≤ 3.81). This index was higher within the Bobo, the Dioula, the Gourmantché, the Kasséna and the Mossi than the Dagara and Fulani (Table 5).

**Table 5:** Comparison of species use values (UVs)

Ethnic group	UVs	*T* test
Bobo	3.81	a
Dagara	3.00	b
Dioula	3.74	a
Gourmantché	3.72	a
Kasséna	3.50	a
Mossi	3.62	a
Fulani	2.80	b

a*b: The levels non-connected by the same letters are significantly different

### Management practices

The individuals of *A*. *africana* were naturally regenerated within the agricultural systems. Practices such as seeding, assisted natural regeneration, or sapling transplantation were absent. However, trees were permanently pruned (32.94 < FL < 76.47) and debarked (23.53 < FL < 50) by local people. Debarking and pruning were mainly practiced by traditional healers and herders to harvest medicinal organs and forage respectively. Pruning was also practiced by farmers to reduce the shade effects on crops productivity (Table 3). Additionally, some trees of the species were systematically eliminated from the farmlands.

### Local ecological knowledge

Informants acknowledged that *A*. *africana* is found in all land use types. More than 50% of informants from most ethnic groups reported *A*. *africana* as a threatened species (51.28 < FL < 100). The protected areas and the sacred groves were cited as areas with high abundance of the species. Several criteria classified into three main groups were recorded regarding local perceptions of variation of *A*. *africana* individuals and populations within and between habitats and land use types. The criteria related to tree structure (size and density) and functional treats (bark texture, bark color, shape of leaves, fruits, and seeds). Besides, "harmful" or taboo trees ("trees which shelter invisible spirits") were enigmatically distinguished from "not harmful" trees ("those which house good spirits"). However, informants did not mention any presence of other *Afzelia* species in their areas.

### Determinants of local knowledge

**Fig. 3:**
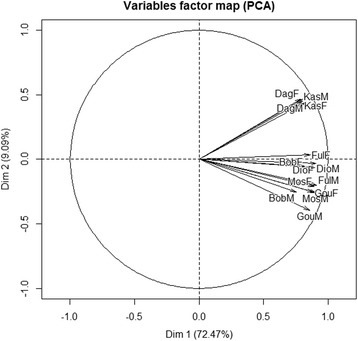
Projection of ethnic-gender groups into the PCA axes. Dim1: principal component 1; Dim2: principal component 2; BobF: female Bobo; BobM: male Bobo; DagF: female Dagara; DagM: male Dagara; DioF: female Dioula; DioM: male Dioula; FulF: female Fulani; FulM: male Fulani; GouF: female Gourmantché; GouM, male Gourmantché; KasF: female Kasséna; KasM: male Kasséna, MosF: female Mossi, MosM: male Mossi

The cumulative percentage of variances derived from the principal component analysis (PCA) revealed that the first two dimensions explain 72.47 and 9.09 of the total variation respectively (Fig. 3). Therefore, these principal components (explaining almost 82% of the total variation) were used to describe the links between people knowledge and their memberships. Figure 3 reveals that all ethnic groups of both sexes are positively correlated with the first axe.

From the correlation matrix (Table 6) and Fig. 3, it appears that women and men from the seven ethnic groups conserved *A*. *africana* for the same motivations (sacred and threatened species, multipurpose uses).

**Table 6:** Correlation of the variables to the axes

Questionnaire item	Variables	PCA 1	PCA 2
Motivation to conserve	Agroforestry species	0.3349	− 0.2913
	Multipurpose species	*0.8952*	− 0.0051
	Sacred species	*0.6105*	− 0.1489
	Threatened species	*0.9710*	− 0.0100
Organs used	Leaves	*0.8700*	− 0.0080
	Barks	*0.7854*	− 0.0777
	Fruits	0.0298	− 0.2844
	Seeds	0.1180	− 0.0008
	Roots	0.0000	− 0.3975
	Wood	*0.6543*	− 0.0141
	Trunk	0.4038	− 0.3732
	Husk	*0.9503*	− 0.0034
Uses in medicine	Headache	*0.6889*	− 0.0089
	Stomach-ache	0.05913	− 0.2962
	Articulations pain	*0.5087*	0.0000
	Teeth pain	0.4472	− 0.0441
	Bilharzia	*0.9678*	− 0.0050
	Hemorrhoids	*0.8704*	− 0.0006
	Meningitis	*0.9774*	− 0.0045
	Malaria	*0.9290*	− 0.0156
	Blindness	*0.9597*	− 0.0001
	Ulcer	*0.9531*	− 0.0032
	Sprain	*0.9706*	− 0.0028
	Sore	*0.6070*	− 0.0647
	Swelling	*0.9540*	− 0.0001
	Sexual impotence	*0.9638*	− 0.0073
	Undiagnosed diseases	*0.9425*	− 0.0044
	Gall	*0.7134*	− 0.0000
	Paralysis	0.9437	− 0.0094
	Mental defect	0.3844	− 0.0003
	Chronic cough	*0.9427*	− 0.0055
	Hard childbirth	*0.9485*	− 0.0012
	Fever	*0.9432*	− 0.0005
	Vertigo	*0.8076*	− 0.0107
Uses in culture	Family protection	0.3373	− 0.1476
	Farm guarding	0.3733	− 0.2412
	Fetish installation	0.3633	− 0.0896
	Anti-bewitching	*0.5173*	− 0.0017
	Magic	*0.5249*	− 0.0835
	Anti-madness	*0.9632*	− 0.0034
	Strength asking	*0.7955*	− 0.0509
	Ask for good season	*0.7438*	− 0.0358
	Twin rituals	*0.6846*	− 0.0642
	Place of sacrifice	0.3285	− 0.0772
	Veneration	*0.8871*	− 0.0231
	Children names	*0.9478*	− 0.0001
	Ask for richness	*0.8699*	− 0.0320
Uses in carpentry	Furniture	0.4243	*0.5093*
	Utensils	0.4521	*0.5137*
	Farming tools	*0.9561*	− 0.0013
	Musical instruments	*0.6554*	− 0.0980
Local practices	Assisted regeneration	0.2394	− 0.0015
	Pruning	*0.8314*	− 0.0140
	Debarking	*0.7582*	− 0.0051
	Fire protection	0.0156	*0.5596*
Occurrence habitats	Farmlands	*0.7358*	− 0.1442
	Fallows	*0.6557*	− 0.2050
	Habitations	0.0002	− 0.0347
	Protected areas	*0.9472*	− 0.0176
	Sacred groves	*0.6038*	− 0.0070
Species status	Abundant	0.0774	*0.6143*
	Rare	0.4504	− 0.0003
Criteria of variations	Tree size	*0.8112*	− 0.0721
	Density	*0.5643*	− 0.0297
	Functional treats	*0.5018*	− 0.0137
Existence of ecotypes	Ecotypes	*0.9601*	− 0.0002
	No other species	*0.9643*	− 0.0022

Values with r ≥ 0.50 are presented in italics

No significant difference was observed among ethnic groups and gender regarding local motivations to conserve the species (Table 7).

**Table 7:** Comparison of people knowledge on *A*. *africana*

Local knowledge	Factor	Degree of freedom	Chi-square	*p* value
Motivation	EG	6	2.847	0.827
	G	1	1.044	0.307
Organs used	EG	6	3.036	0.804
	G	1	0.031	0.860
Food uses	EG	6	8.247	0.220
	G	1	0.027	0.870
Medicinal uses	EG	6	17.904	0.006*
	G	1	8.280	0.004*
Fodder use	EG	6	6.000	0.423
	G	1	0.125	0.723
Cultural use	EG	6	12.723	0.048*
	G	1	6.318	0.012*
Handicraft use	EG	6	5.644	0.464
	G	1	0.892	0.345
Local practices	EG	6	9.026	0.172
	G	1	0.558	0.455
Occurrence habitat	EG	6	13.233	0.039*
	G	1	0.000	0.977
Species status	EG	6	5.083	0.533
	G	1	0.256	0.613
Perception of variation	EG	6	5.055	0.536
	G	1	1.005	0.315
Existence of ecotypes	EG	6	0.000	1.000
	G	1	0.048	0.826

*EG* ethnic group, *G* gender

*Significant differences at *p* < 0.05

The variables leave, wood and bark positively correlated with the principal component 1 (Table 6) represent the main used products. These products are used to address 20 diseases and to fulfill 10 magico-religious practices. The medicinal uses of the species varied significantly between ethnic groups (*p* = 0.006) and gender (*p* = 0.004) (Table 7). Similarly, significant differences were observed between ethnic groups (*p* = 0.047) and gender (*p* = 0.012) regarding the cultural uses.

The species was found in all land use types with significant differences among ethnic groups regarding the occurrence habitats (*p* = 0.039). However, informants perceived the species rarer in their areas and observed variations in tree size, density, and functional treats. Assisted natural regeneration of *A*. *africana* was absent. However, local people practiced debarking and pruning. The second axe separates the Dagara and Kasséna of both sexes from the five other ethnic groups. Both genders of the Dagara and Kasséna ethnic groups are highly correlated with the principal component 2, with positive correlation values (*r* ≥ 0.5) in contrast to the other ethnic groups. From this second principal component, it appears that male and female Dagara and Kasséna used *A*. *africana* in carpentry, found its populations abundant in their areas, and protected the species from fire.

## Discussion

### Importance of *A*. *africana* to rural communities

*A*. *africana* is well known by rural communities who identify it through different names. Nevertheless, some names are phonetically similar between ethnic groups. This involves the denominations Kakala, Kankalga, and Kakalgahi respectively from the Dagara, Mossi and Fulani, and Linguè, Linguèhè from the Dioula and Fulani. Overall, these names referred to the inhabitants of the species that is locally perceived as a taboo or magical tree [17]. For instance, the name "Kontontiè" in Dagara means dwarfs or evils whereas "Bunakpambu" in Gourmantché refers to shrines, fetishes, or sanctuaries. Through these names, *A*. *africana* is considered as a taboo or magico-mystic tree sheltering invisible forces, as reported on *Sterculia setigera* Delile in Togo [30]. The mystery and the fear linked to the invisible forces and the species multipurpose uses justify its conservation within the agroecosystems.

This study demonstrated that rural Burkinabès have good knowledge on the sociocultural value of the legume tree *A*. *africana*. Therefore, it  contributes to preserving traditional knowledge on wild plant uses, as a basis to guiding conservation actions. The results clearly indicated that *A*. *africana* is a multipurpose agroforestry species and confirmed several studies across West Africa [2, 11, 13, 23]. The species is widely used by rural communities in livestock breeding, food, pharmacopeia, culture, construction and carpentry. Animal fodder and traditional medicine constituted the first two most important uses of this Fabaceae species, corroborating Zizka et al. [5] who found that the medicinal and cultural uses were the most important within the Fabaceae plants in Burkina Faso. The leaves are used to feed cattle during the dry season in the Sahelian countries where forage scarcity severely handicaps animal breeding. The high fodder value of *A*. *africana* leaves highlighted by informants also corroborates studies conducted in Southwestern and Western Burkina Faso [13, 15]. The leaves are also used as vegetables by some locals [2, 19].

The medicinal uses were the most diversified with more than 20 diseases addressed with *A*. *africana* organs. This is consistent with other authors [1–3, 5] who highlighted the importance of traditional medicine to rural peoples in Burkina Faso. In fact, most peoples living in rural areas strongly rely on many medicinal plants for their healthcare. The barks were reported to be highly medicinal, as confirmed by prior studies [11, 12, 16]. The decoction and/or the powder of all tree parts (barks, leaves, roots, seeds, and husks) were recognized as being efficiently used to address several infections, as also indicated by Orwa et al. [12].

The magico-mythical properties of *A*. *africana* as also reported [12, 17] make this species very useful in magico-religious fulfilments such as farmlands, human, or families protecting against invisible forces, wizards, and enemies. Considered as fetish, *A*. *africana* trees and organs are used to fulfill people spiritualities such as achieving crop productivity, power, fecundity, and richness. These cultural uses of plant resources have been largely quoted in Burkina Faso [4], in Togo [30], and in Benin [31], revealing the links between mankind culture and plants. The sacred or taboo plants are more used in cultural fulfilments [32]. Considering all the medico-magical usefulness of *A*. *africana*, it is important to convince local people towards its protection.

The wood was cited as a good source for various craft items and was traditionally used for manufacturing pickaxes, mortars, pestles, spatula, traditional guns, canoe and djembé. These results are in accordance with other studies [11, 12] which showed that *A*. *africana* wood is termite resistant making it highly commercial and excellent for various industrial purposes. These good mechanical properties of the wood [11] confer high resistance and long life to the derived craft items. However, *A*. *africana* wood was forbidden from being used as firewood within some families and ethnic groups. Similar results were reported on other sacred plants such as *Tamarindus indica* L. and *Diospyros mespiliformis* Hochst [16, 17]. In fact, local trees protected by traditional bans are not burnt [1, 3, 30] because of people beliefs on the enigmatic embodiments of such plants. The burning of these taboo plants would expose the offenders to unpredictable fates. The use in energy of *A*. *africana* wood by a pregnant woman for instance may result in bad spells, blindness, madness, or devil birth. Such local beliefs corroborate Korbéogo [32] who mentioned that the burning of the Gourmantché sacred plants induces within this ethnic group the manifestation of evil spirits and unlucky events. These traditional bans and beliefs on the nature contribute in a certain measure to protect plant species.

Apart from the uses of *A*. *africana* reported in this study, other properties of this species were reported elsewhere in Africa. *A*. *africana* seeds are consumed by people as a soup thickening agent in Nigeria [12]. It has been proven that these seeds possess extractable oil usable in biofuel production [33]. Furthermore, the seeds’ oils are nutritionally useful and possess various household uses [34] and industrial applications [12]. The study reported a traditional use of *A*. *africana* wood in Burkina Faso. However, *A*. *africana* wood called "doussié" has great economic and industrial values elsewhere in Africa [11]. In fact, *A*. *africana* (Sm.), *A*. *bella* (Harms), *A*. *bipindensis* (Harms), and *A*. *pachyloba* (Harms) are the four African *Afzelia* species traded internationally with Cameroon as the main exporting country [11, 35]. The average price of *Afzelia* sawn wood is US$ 780 per m^3^ and the other main exporters in Africa are Ghana, Côte d’Ivoire, Congo, and Gabon [35]. The promotion of these properties in Burkina Faso may motivate local people to domesticate the species. This might contribute to enhance local incomes that constitutes the main challenge of the sustainable development goals.

### Distribution of use values

The findings showed that the usefulness of *A*. *africana* differs according to the ethnic groups. In fact, the use values revealed that the Bobo, Dioula, Gourmantché, Kasséna (natives), and Mossi (migrants) ethnic groups had more knowledge and more uses on *A*. *africana* compared to the Dagara (natives) and Fulani (migrants) who displayed the lowest use values. Such variations could be explained by differences in people cultural background, as highlighted by other ethnobotanical findings [3, 26, 27]. The species underuse by the Dagara could be ascribed to their exaggerated magical perception that limits the species uses. The main use of *A*. *africana* as fodder by the Fulani might explain the low use value observed within this ethnic group. This specific fodder use is consistent with the Fulani tradition as herders. The random distribution of the use values among autochthons and migrants suggest that the utilitarian value given to a species by local people does not depend on their residence status. Therefore, both natives and migrants may be knowledgeable or not on plant uses, depending on people cultural backgrounds and plant distribution range.

### Local management strategies

Compared to other agroforestry species such as *Parkia biglobosa* (Jacq.) R.Br. ex G.Don, *Vitellaria paradoxa* C.F.Gaertn., and *Tamarindus indica* L. which benefit from particular management practices such as assisted natural regeneration, seeding or often sapling transplantation within the farmlands, *A*. *africana* seems to be neglected. This might be due to the magic or taboo status of the species. In fact, the myth or taboo associated with *A*. *africana* constitutes big challenges towards its domestication and sustainable conservation. Although local perception on the sacred or taboo plants are indispensable supports for their conservation [36], the prejudices linked to their inhabitants prevent some farmers to maintain them in their farmlands or to assist their regeneration. The ignorance of the economic value of *A*. *africana* in the local areas might also explain this lack of local management strategies. The trees of *A*. *africana* were severely pruned and debarked. Informants assumed that foliage harvesting rejuvenates the trees and renews the foliage. These perceptions are consistent with other findings that showed that pruning of *Khaya senegalensis* (Ders.) A.Juss. improves the quality and quantity of leaves produced [37]. However, the harvesting of barks was perceived by locals as being more prejudicial to the species survival. Indeed, debarking exposes the stem to desiccation, fires, drought and various attacks [38]. It is recognized that harvesting of bark has more negative impacts on the habitat and populations of plant species than leaves and fruits harvesting [27]. Severe pruning weakens the tree [37] whereas severe debarking induces tree mortality in the long term. The combined bark and foliage harvesting decreases the production of fruits and seeds of *A*. *africana* [38].

### Ecological knowledge on *A*. *africana*

Due to the absence of regeneration practices and the unsustainable exploitation of *A*. *africana*, its populations were acknowledged declining in the rural areas across all study sites. This result concurs with other findings in Burkina Faso and Benin where the species is acknowledged threatened [14, 22] and endangered [20, 21] respectively. The severe pruning and debarking might explain the lower density of the species within the agricultural systems. These variations in tree structure across land use types as reported by locals corroborate findings that indicated the negative effect of land use on the dynamics of *A*. *africana* in West Africa [14, 20, 21]. The protected areas that represent the latest refuges for many threatened animals and plants [39] were quoted as principal refuges for *A*. *africana*.

In contrast to local perceptions on the existence of morphotypes within *A*. *africana* species, no existence of other *Afzelia* species was mentioned. These observations are congruent with scientific data supporting that among the seven African *Afzelia* species, only *A*. *africana* is found in Burkina Faso [11]. Nevertheless, the morphological traits reported on the species raise a need for further molecular studies and thorough botanical researches able to testify the occurrence of this unique African *Afzelia* species in Burkina Faso.

### Driving factors of local knowledge

The motivations to conserve *A*. *africana*, the species uses, the parts exploited, and the occurrence habitats showed a homogeneous distribution among all ethnic groups of both sexes. However, in contrast to the other ethnic groups, the Dagara and Kasséna of both sexes perceived *A*. *africana* abundant in their areas. Such  results imply similar global trends in local knowledge of plants across communities on the one hand, and specific cultural habits on the other hand. Indeed, people of different cultural backgrounds might share global similar customs with some variations across communities due to their specific culture. Significant differences were observed between ethnic groups regarding the species medicinal and cultural uses. These variations corroborate other ethnobotanical findings in West Africa that showed the influence of ethnicity on plant uses [3, 10, 25, 27]. This might explain the high diversity of the medicinal and cultural uses of *A*. *africana*. The medicinal and cultural uses varied also according to gender, as corroborated by studies on other plants [10, 27]. However, these differences observed between men and women disagree with studies which demonstrated that gender is not a determinant of plant uses in Burkina Faso [2, 3, 25]. These disagreements might be due to the magical embodiments of *A*. *africana* making men more knowledgeable on the species uses than women. Significant differences were also observed between ethnic groups regarding the species habitats. The variability in local ecological and environmental conditions controlling the species distribution and availability [3] could explain such differences between ethnic groups regarding local perceptions on the occurrence habitats and species abundance.

### Implications for sustainability

The present study provides insight into the use patterns and management of African mahogany, and their impacts on the species spatial distribution, as a basis to set up an efficient sustainable conservation plan. The findings provide relevant social and ecological indicators likely to guide local people and decisions’ makers towards the species sustainability. The multipurpose uses of *A*. *africana* reported as the main reason for its in-situ conservation indicate that promoting its domestication will be welcomed by local people, especially the Bobo, Dioula, Gourmantché, Kasséna, and Mossi ethnic groups who gave high utilitarian value to the species. Similarly, considering the recent forage shortage in the Sahel region and the contribution of animal breeding to rural incomes, the high fodder and veterinary value of *A*. *africana* leaves reported in this study is a key indicator to promote the species domestication. The diverse medicinal properties of *A*. *africana* offer opportunities for pharmacological researches.

Given its wide distribution range [11, 12] and its wide adaptation to climatological conditions [35], *A*. *africana* can be promoted in regional programs of restoration that is a major issue in West Africa under increasing land degradation. With the high economic value of *A*. *africana* wood in Africa [11, 35], promoting its use in national programs of domestication and restoration could increase doussié production and generate important economic incomes. Such programs could also contribute to climate change mitigating via carbon sequestration. Although the seeds’ oils of *A*. *africana* were reported to be potentially useful for bioenergy production [33], the seeds were neglected and rarely used by rural communities. Therefore, their vulgarization might boost its seedling plantation and capture the attention of global bioenergy market towards the species sustainable conservation.

People perceptions that *A*. *africana* houses evil-doing spirits might constitute big challenge to meet its domestication. These negative perceptions imply sensitizations that may convince local people to assist the species natural regeneration or to maintain its individuals within the farmlands. The education of local people towards sustainable practices such as soft harvesting patterns could boost a rational use of the species and its sustainable conservation. In this perspective, avoiding debarking or reducing its intensity and frequency is strongly recommended.

The perceptions of rural communities on the habitats and the dynamics of *A*. *africana* reveal the double function of hotspots and refuges, attributable to the protected areas [39]. Indeed, the protected zones greatly contribute to protecting the threatened species from extinction. These ecosystems remain the latest refuges for many threatened species. More investigations are highly required to explain local perceptions on the diversity of tree functional traits and their variations across individuals and habitats.

Gender and ethnicity aspects should be considered in domestication and sustainable management projects of high-valued tree species such as *A*. *africana*.

## Conclusion

This study confirmed that *A*. *africana* is a threatened and multipurpose agroforestry tree extensively exploited throughout Burkina Faso. The use values of the species varied according to people cultural backgrounds with the Dagara and Fulani having the least level of knowledge. Despite its multipurpose uses, *A*. *africana* is neglected within the farmlands with a quasi-total absence of management practices. The harvesting of barks as practiced by traditional healers impacted negatively on the species survival. Local people perceived declined populations of *A*. *africana* especially in the farmlands. The study also showed that these communities perceived variations in the targeted species populations' structure and trees morphological traits. The uses of *A*. *africana* were influenced by gender and ethnicity as well. However, only ethnicity affected people knowledge on the species habitats. The study revealed strong concordance between indigenous knowledge and scientific findings. While the results provide relevant indicators likely to guide conservation decisions, local magical beliefs might constitute severe challenge to domesticate African oak in Burkina Faso.

## Abbreviations

FL: Fidelity level
IFS: International Foundation for Science
IUCN: International Union for Conservation of Nature
IVO: Index value of organs
PAs: Protected areas
PCA: Principal component analysis
UVs: Use value of the species
WASCAL: West African Science Service Center on Climate Change and Adapted Land Use
